# Oral health related-quality of life before and during the COVID-19 pandemic in patients with non-alcoholic fatty liver disease

**DOI:** 10.4317/medoral.25731

**Published:** 2023-01-15

**Authors:** Inácio Lima Silva Aguiar, Fernando M Carvalho, Larissa Souza Santos-Lins, Rebeca Brasil-Oliveira, Carlos Brites, Helma Pinchemel Cotrim, Jerry E Bouquot, Liliane Lins-Kusterer

**Affiliations:** 1 Programa de Pós-graduação em Medicina e Saúde, Faculdade de Medicina da Bahia, Universidade Federal da Bahia, Salvador, Bahia, Brazil; 2 Graduate Program in Medicine and Health, Bahia School of Medicine, Federal University of Bahia, Salvador,Bahia, Brazil

## Abstract

**Background:**

Since the beginning of the COVID-19 pandemic, the number of medical appointments and the offer and use of oral health services have decreased sharply with the lockdown period. Restriction to regular dental care can increase the risk of oral diseases, capable of affecting general health and oral health-related quality of life, particularly among medically compromised patients. This study aimed to assess health-related quality of life (HRQoL) and oral health-related quality of life (OHRQoL) of patients with non-alcoholic liver disease (NAFLD) before and during the COVID-19 pandemic.

**Material and Methods:**

Prospective cohort of 58 patients with NAFLD followed up from March 2020 (before the pandemic) to December 2021 (during the pandemic). RAND 36-Item Health Survey and Oral Health Impact Profile 14 (OHIP-14) questionnaires were used to assess HRQoL and OHRQoL, respectively, in the two points of time.

**Results:**

The scores of all scales HRQoL and of the question about health change in the last year decreased substantially with the advent of the pandemic. Large (>0.50) effect sizes were estimated for the scales Role functioning/physical, Pain, General health, and Energy/fatigue. Patients who had COVID-19 presented better HRQoL and OHIP-14 mean scores than those who did not have the disease. The OHIP-14 total score increased 3.6 points with the advent of the pandemic, representing a large effect size (0.62). Patients presented high probability (84.3%) of increasing OHIP14 score during the pandemic.

**Conclusions:**

The HRQoL and the OHRQoL scores of NAFLD patients decreased substantially with the advent of the pandemic. However, these decreases were not associated with the COVID-19 disease by itself, but probably to other factors related to the deep social changes brought by the social isolation measures to combat the pandemic.

** Key words:**Non-alcoholic fatty liver disease, health-related quality of life, oral health, COVID-19.

## Introduction

The COVID-19 pandemic had deep impact on the health services, all over the world. In Brazil, the first case of COVID-19 was reported on 26 February 2019 (1). The number of medical appointments and the offer and use of oral health services have decreased sharply with the lockdown period (2,3). Restriction to regular dental care can increase the risk of oral diseases, such as dental caries, periodontal disease, soft tissues lesions, and temporomandibular joint disorders, capable of affecting general health and oral health-related quality of life (OHRQoL) (4).

Non-alcoholic fatty liver disease (NAFLD) is currently recognized as the most common chronic liver disease, with an estimated prevalence, diagnosed by imaging, of 25.2% (95% CI, 22.1-28.7), according to a meta-analysis (5). NAFLD has many risk factors, including insulin resistance, obesity, diet, obstructive sleep apnea, genetic factors, and infection by certain types of intestinal bacteria (6). In addition, oral infection by *Porphyromonas *gingivalis**, a major causative agent of periodontitis, has been associated with NAFLD (7,8). The poor oral health status observed in most chronic liver disease patients may represent a source of systemic infections (9), leading to poor overall health-related quality of life (10).

This study aimed to evaluate health-related quality of life (HRQoL) and oral health-related quality of life in patients with non-alcoholic liver disease, before and during the COVID-19 pandemic period.

Material and methods

- Study design and population

This is a prospective cohort investigation of health-related quality of life and oral health-related quality of life in 58 patients with NAFLD attending the Hepatology Outpatient Clinic of the University Hospital of Federal University of Bahia, Brazil. All patients attending the Clinic from February 2018 to February 2020 were invited and all agreed to participate. From March 2020 onwards, they were followed up by telehealth. There were no refusals to share the study nor losses of follow up, probably because good, free dental care is not available to these patients in the state of Bahia.

Data collection was performed twice: prior to the pandemic information was collected in a structured questionnaire about sociodemographic (age, sex, ethnicity, civil status, monthly family income), practice of physical activity, grade of NAFLD, time since NAFLD diagnostic, metabolic syndrome, habits of oral hygiene (teeth brushing frequency, and dental flossing). Oral examinations were performed according to criteria from the World Health Organization and the European Association of Dental Public Health (11,12), including the assessment of decayed, missing and filled teeth (DMFT index). Salivary flow was collected as described elsewhere, with reduced flow defined as values less than or equal to 1 mL/min (10). Oral Health Impact Profile 14 (OHIP-14), and RAND 36-Item Health Survey questionnaires were used to assess HRQoL and OHRQoL, respectively.

The second evaluation was undertaken during the COVID-19 pandemic, with data collection occurring from May 2021 to December 2021. Using telehealth, OHIP-14 and RAND-36 questionnaires were applied again. Patients were asked about the occurrence of caries, gum inflammation, toothache, tooth fracture, temporomandibular joint pain, and COVID-19, during the period 1 March 2020 to 31 December 2021.

- OHIP-14

The Oral Health Impact Profile 14 (OHIP-14) was used to assess the OHRQoL. Its 14 items are divided into seven domains: functional limitation, physical pain, psychological discomfort, physical disability, psychological disability, social disability, and handicap. Answers are classified in Likert scale and coded as 0 = “never”, 1 = “hardly never”, 2 = “occasionally”, 3 = “fairly often” and 4 = “very often/every day”. Domain scores can range from 0 to 8. The OHIP-14 score can range from 0 to 56 and calculated by summing the values for the 14 items. Higher values mean poorer OHRQoL (13).

- RAND 36-Item Health Survey

The RAND-36 is a questionnaire with 36 items that generate eight health-related quality of life scales: physical functioning (10 items), role limitations due to physical health (4 items), bodily pain (2 items), general health (5 items), energy/fatigue/vitality (4 items), social functioning (2 items), role limitations due to emotional problems (3 items), emotional well-being/mental health (5 items). Scores can range from 0 to 100, and higher scores mean good health-related quality of life. Question 2 of the RAND-36 questionnaire measures health change over time, by asking: "Compared to one year ago, how would you rate your health in general now?” Possible answers were: 1 - Much better now than one year ago; 2 - Somewhat better now than one year ago; 3 - About the same; 4 - Somewhat worse now than one year ago; 5 - Much worse now than one year ago. Answers were converted into scores ranging from 0 to 100, with higher scores meaning a change towards better health (14). The Brazilian version of the RAND-36 questionnaire was validated in a study of 783 individual, 400 (52.4%) with chronic liver disease. Confirmatory factor analysis revealed good indices of adherence to the model with eight scales and internal consistency, measured by the composite reliability index, was high (14).

- Statistical analysis

Statistical analyses were performed by using the Statistical Package for the Social Science (SPSS) version 21 (IBM Corporation, Armonk, NY, USA). The internal consistency of the RAND-36 scale and of the OHIP-14 total score was assessed by Cronbach’s alpha, the values of which were considered satisfactory at 0.70-0.80 and ideal at 0.80-0.90 (15).

Bivariate analyses used the Wilcoxon nonparametric test for related samples to compare OHIP-14 and RAND-36 scores, and health changes measured before and during the pandemic. Effect size was evaluated by Cohen’s “r” obtained by dividing the Z statistics provided by the Wilcoxon test by the square root of the sample size (r = Z / √N). The value of “r” was interpreted as small (0.10 to 0.30); moderate (0.31 to 0.50); and large (>0.50) (16). The Common Language Effect Size (CLES), also known as probability of superiority (17), provides an estimate of the probability that, in a randomly selected pair of scores of a same patient, the score at the “During” moment be lower than the score at the “Before” moment. CLES was calculated by dividing the number of positive difference scores by the total number of matched pairs, and discarding eventual ties (16). Fifty per cent would be a “neutral” value (18).

Bivariate analyses used independent sample t-tests or Pearson correlation in the case of continuous variables (sex, mean family income, time since NAFLD diagnostic, and DMFT index) to compare “During - Before” mean values of RAND-36, health change in the last year, and OHIP-14 index according to the strata of the variables of interest.

Variables associated at p≤0.20, in the bivariate analyses, with the outcomes (RAND-36 scales, health change in the last year, and OHIP-14 index) were select to compose the respective ten multiple regression models. The variable COVID-19 was forcibly included in all models, irrespective of its P-value. The selected variables were inserted as a block in each equation, using the default selection method ‘Enter’. Cases presenting studentized residual analysis varying ± 3.000 standard deviations were identified as outliers.

- Ethical aspects

The study was approved by the Ethics Committee of the Faculty of Medicine of the Federal University of Bahia (protocol (number 2.780.060), in accordance with Resolution 466/2012 of the Brazilian National Health Council, and the Declaration of Helsinki 1975, revised in 2013.

## Results

Before the COVID-19 pandemic, the study population of 58 patients diagnosed with non-alcoholic fatty liver disease had a mean age of 56.1 ± 10.5 years, with a female predominance (79.3%), Afro-American predominance (82.8%), and a predominance of singles (53.47%); 56.9% practiced regular physical activity. The mean monthly family income was R$ 2,498 ± 1,200 (equivalent to US$ 681 ± 327), ranging from R$ 960 to R$ 4,500 (US$ 262 to 1,226). Thirty-eight (65.5%) patients had a non-alcoholic fatty liver disease grade of 2 or higher; the mean time since diagnosis was 77 ± 3 months, ranging from 3 to 348, and 28 (48.3%) presented with metabolic syndrome. The DMFT index (20.4 ± 7.9) was high, particularly because of high numbers of missing teeth (15.7 ± 9.1); there was a high percentage of patients with reduced salivary flow (65.5%), and high prevalence of gingivitis (53.4%), and periodontitis (31.0%). Brushing teeth more than twice a day and using dental floss was reported by 44.8% and 48.3% of the subjects, respectively (Table 1). There were no actual smokers or drinkers in the study population.

**Table 1:**
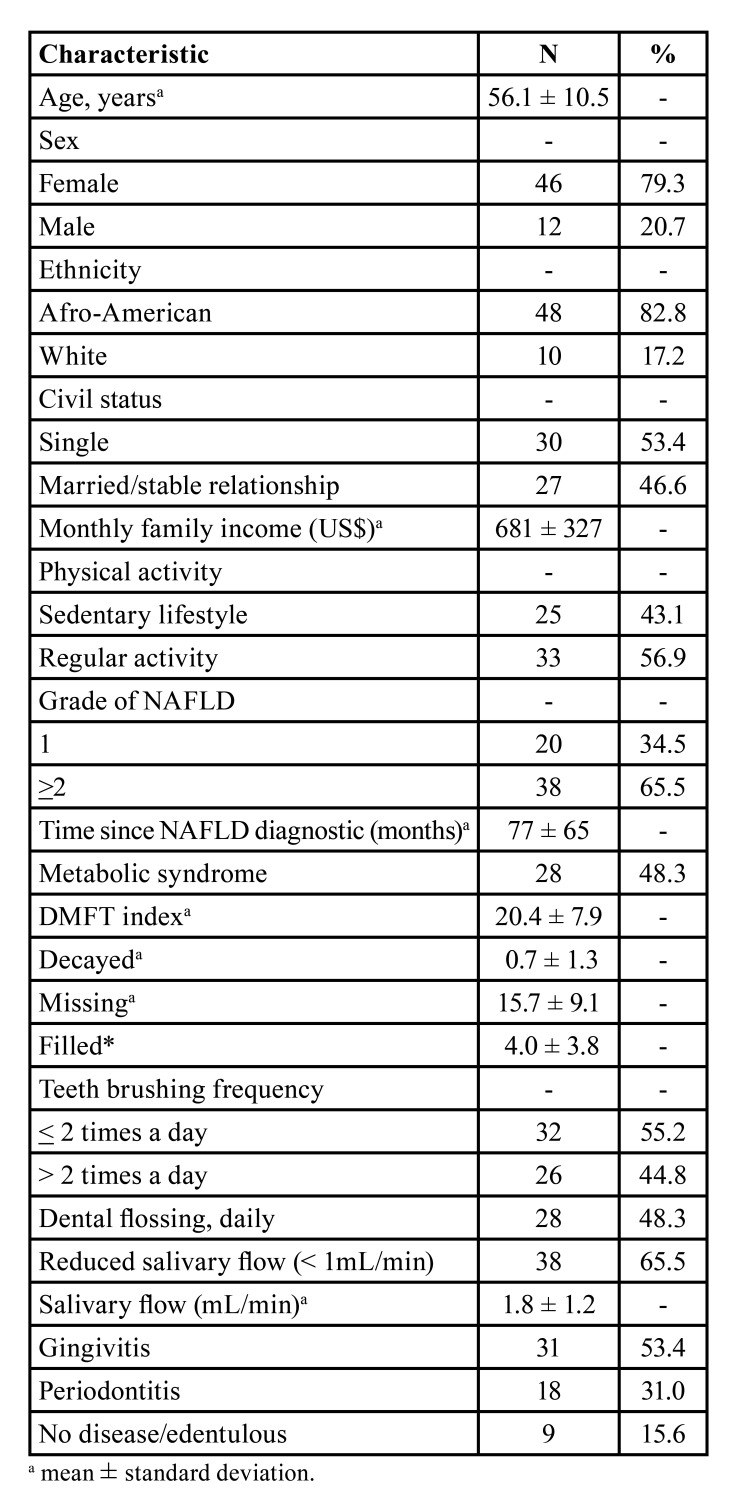
Demographic, clinical and oral health characteristics of 58 individuals with non-alcoholic fatty liver disease, Salvador, Brazil, before the COVID-19 pandemic, February 2018 to February 2020.

During the COVID-19 pandemic, from March 2020 to December 2021, 27 (46.6%) of the 58 subjects referred they had COVID-19. Two subjects were hospitalized because of COVID-19. Overall, the subjects reported caries (58.6%), gum inflammation (60.3%), toothache (32.8%), tooth fracture (5.2%), and temporomandibular joint pain (36.2%).

The scores of all scales of the health-related quality of life and the score of the question about health change in the last year decreased substantially with the advent of the COVID-19 pandemic. Large (>0.50) effect sizes were estimated for the scales Role functioning/physical, Pain, General health, and Energy/fatigue. A decrease of 32.8 points in the Role functioning/physical scale was noteworthy. The common language effect size for Role functioning/physical scale indicated that 94.6% of subjects presented “During” scores lower than their respective “Before” scores, excluding those who presented the same value at “During” and “Before” measurements. Cronbach’s alphas were above 0.80 in seven scales, and 0.70-0.80 in one scale, both before and during the pandemic. The OHIP-14 total score increased 3.6 points (*p*<0.001) with the advent of the pandemic, representing a large effect size (0.62) and high probability of superiority, representing that 84.3% of the patients had increased the OHIP14 score during the pandemic. Cronbach’s alphas for OHIP-14 total scores were high before (0.853) and during (0.882) the pandemic (Table 2).

The “During – Before” mean scores of Physical functioning, Role functioning/physical, Pain, General health, Energy/fatigue, Emotional well-being, the health change in the last year, and OHIP-14 were worse among the 31 subjects who did not report having COVID-19 than among those 27 who reported having COVID-19. By their turn, Social functioning and Role functioning/emotional scales presented worsening in the “During – Before” mean scores among patients who did report having COVID-19 (Table 3).

Eight out of nine linear multiple regression equations revealed that the variable COVID-19 was positively associated with variations in the scores of HRQoL scales and health changes in the last year, after controlling for other variables selected by previous bivariate analysis. In other words, patients with COVID-19 presented higher health-related quality of life than patients who did not report the infection. An exception was the Social functioning scale, which was negatively associated with COVID-19: there was an equation-estimated 5.026-point decrease in the mean score of this scale among subjects with a positive COVID-19 history, compared to patients who did not report the infection. However, this difference was not statistically significant (*p*=0.102) (Supplement 1).

The oral health-related quality of life, measured by the OHIP-14 total score was not strongly associated with a positive COVID-19 history, in the multiple linear regression model. However, the model estimated OHIP-14 scores 6.689 units lower (*p*<0.001) for patients who reported toothache and 7.151 units lower (*p*=0.002) for those reporting tooth fracture. Residual analysis revealed two individuals with high studentized residues (3.364 and -3.315 standard deviations, respectively); these were excluded from the model. The model showed good adherence to the data, as revealed by a high adjusted R2 = 0.523, and an accepTable value for the Durbin-Watson statistics (=2.420). Collinearity was negligible, as revealed by tolerance collinearity statistical (1 - R2) values ranging from 0.634 to 0.943. Tolerance statistics close to zero indicated that a variable was almost a linear combination of the other predictors in the model. Adjusted R2 was 0.523 and a Durbin-Watson statistics equal to 1.475 fell within the accepTable range (1.5 to 2.5). Small numbers in many strata prevented drawing conclusions about interactions among the predictors investigated (Table 4).

**Table 2:**
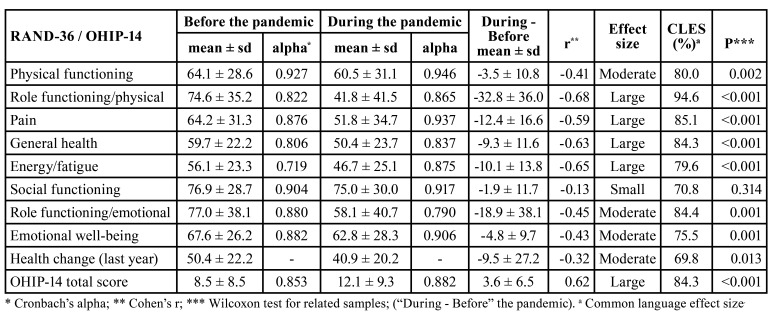
Health-related quality of life (RAND-36) and OHIP-14 scores of 58 individuals with non-alcoholic fatty liver disease before (February 2018 to February 2020) and during (March 2020 to December 2021) the COVID-19 pandemic, Salvador, Brazil.

**Table 3:**
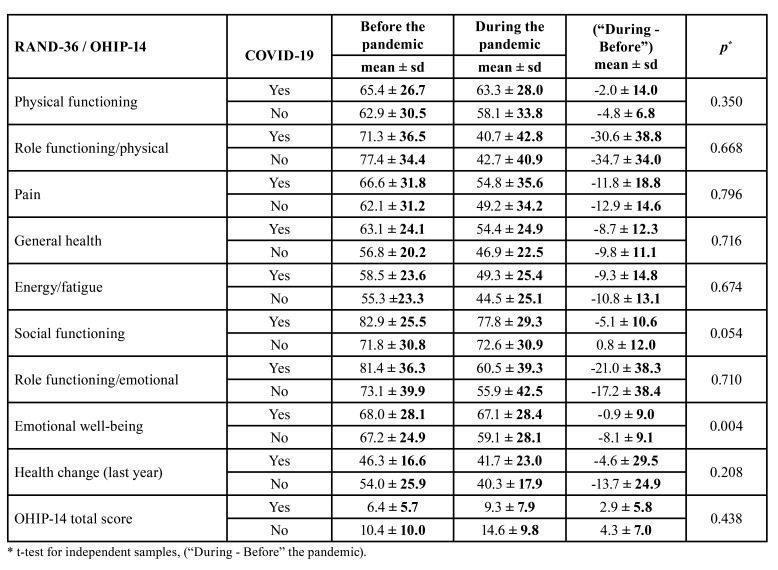
Health-related quality of life (RAND-36) and OHIP-14 scores of 58 patients with non-alcoholic fatty liver disease according to infection by COVID-19 before (February 2018 to February 2020) and during (March 2020 to December 2021) the pandemic, Salvador, Brazil.

**Table 4:**
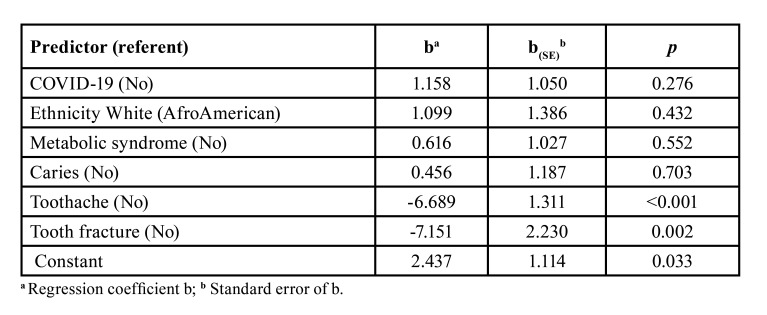
Multiple linear regression equation having OHIP14 “During - Before” score (%) as dependent variable in 56 patients with non-alcoholic fatty liver disease.

## Discussion

The key results of this study were the remarkable decreases in the health-related quality of life and in the oral health-related quality of life scores of patients with non-alcoholic liver disease associated with the advent of the COVID-19 pandemic. These decreases could not be attributed to the COVID-19 disease by itself, and so probably resulted from the deep social changes brought by the social isolation measures initiated to combat the pandemic.

During the pandemic, emergency health services were prioritized, with both elective care and preventive screening for disease diminished in status in order to reduce the exposure of health professionals and patients to the new coronavirus and to avoid the overloading of health systems (2,19). As a result, patients with a variety of chronic diseases were not followed up, which had a significant impact on HRQoL and OHRQoL. The pandemic appears to have been an important factor in the decrease in HRQoL (20), since there was a potential emergence of disorders related to traumatic stress. This may explain the significant drop that occurred in all scales of the questionnaires applied during our study. The same reasoning applies to the decrease observed in the oral health-related quality of life.

The mean scores of all eight scales and the health change question of the RAND-36 questionnaire decreased substantially, and the OHIP-14 score increased, presenting moderate and large effect sizes. Another approach to evaluating these changes would include the Minimal Clinically Important Difference (MCID), defined as the smallest difference in score in the scale of interest which is perceived to be beneficial or harmful, and that would imply the need for change in a patient´s management (21). A 5.0-point value has been adopted for all domains and the health change question in investigations of individuals with moderate to severe psoriasis, using RAND-36 and SF-36 (22,23). MCID values are very variable, depending on methods used in its calculation and on the clinical context of the study (24). It is recommended that the MCID should be ascertained for each particular study population (25).

To the best of our knowledge, the MCIDs of the RAND-36 scales and of OHIP14 have not yet been determined for patients with NAFLD. However, differences as great as those found in our “During - Before” mean scores for Role functioning/physical (-32.8 points) and Role functioning/emotional (-18.9 points) and in OHIP14 (3.6 points) were remarkable and leave no doubt about the magnitude of these decreases after the advent of the COVID-19 pandemic. Further, it must be kept in mind that the MCID for the group-level is necessarily smaller than for the individual patient-level, because of greater measurement errors inherent to patient quality of life scores (26).

In this study, patients who had COVID-19 presented similar or even better HRQoL scores than those who did not have the disease. as revealed by bivariate and multivariate analyses. Despite the two hospitalized COVID-19 cases, this finding suggests that other factors were more important than this disease in determining the decrease in health-related quality of life of persons with NAFLD.

The oral health-related quality of life of these NAFLD patients has also worsened during the pandemic. Bivariate analyses found an increase of 3.6 ± 6.5 points in the OHIP-14 “During - Before” mean score. But, after controlling for other relevant variables, including COVID-19, a multivariate linear regression model estimated that this decrease could be as large as -7.151 ± 2.230 for patients who reported tooth fracture and -6.689 ± 1.311 for patients who reported toothache during the pandemic. This analysis reinforces the idea that other factors are more influent to oral health-related quality of life than the disease COVID-19. The 88% decrease in the number of dental care procedures in 2020, compared with 2019, in the 5,544 Brazilian municipalities may be largely responsible for this but the present investigation could not determine that (27).

The period covered by this study was particularly hard for the Brazilian health system, due to the COVID-19 pandemic. By the time of this study, 22,291,839 cases and 619,334 deaths due to COVID-19 were notified in Brazil by the Coronavirus Resource Center of the Johns Hopkins University of Medicine (coronavirus,jhu.edu/region/brazil). Brazil responded by approximately 11.5% of the 5.420,000 deaths due to COVID-19 in the world (https://www.who.int/data/stories/global-excess-deaths-associated-with-covid-19-january-2020-december-2021), inspite of having only 2.7% of the global population (https://countrymeters.info/en/World#:~:text=Demographics%20of%20the%20World%202021&text=This%20is%20an%20increase%20of,of%207%2C851%2C163%2C856%20the%20year%20before).

This study has a few limitations that may affect the validity of the conclusions, such as its small sample size, the unknown nature of referral biases in health clinic patients, and the inherent limitations of observational studies. Additionally, the final data collection was performed by teleconsultation and, therefore, all obtained data were, by necessity, subjective, while much of the original data was based on clinician observation. However, this potential measurement bias was most likely minimized, since internal consistency of the questionnaires was satisfactory, considering that the Cronbach’s alpha coefficients remained always high before and during the pandemic.

In conclusion, this study has determined that the COVID-19 pandemic had strong negative impacts on the health-related quality of life and on the oral health-related quality of life of patients with NAFLD. Other factors brought by the pandemic (lockdown, social isolation, and the restricted access of patients to face-to-face health services), rather than infection by the coronavirus itself, appear to be responsible or more impactful.
